# Dynamical model fitting to a synthetic positive feedback circuit in *E. coli*

**DOI:** 10.1049/enb.2020.0009

**Published:** 2020-06-23

**Authors:** Jure Tica, Tong Zhu, Mark Isalan

**Affiliations:** ^1^ Department of Life Sciences Imperial College London London SW7 2AZ UK

**Keywords:** differential equations, time series, biology computing, feedback, genetics, circuit feedback, dynamical model fitting, synthetic positive feedback circuit, synthetic biology, modular genetic components, positive feedback circuit, phage shock promoter, ordinary differential equation model, time‐series data, complex dynamics, E. coli

## Abstract

Applying the principles of engineering to Synthetic Biology relies on the development of robust and modular genetic components, as well as underlying quantitative dynamical models that closely predict their behaviour. This study looks at a simple positive feedback circuit built by placing filamentous phage secretin pIV under a phage shock promoter. A single‐equation ordinary differential equation model is developed to closely replicate the behaviour of the circuit, and its response to inhibition by TetR. A stepwise approach is employed to fit the model's parameters to time‐series data for the circuit. This approach allows the dissection of the role of different parameters and leads to the identification of dependencies and redundancies between parameters. The developed genetic circuit and associated model may be used as a building block for larger circuits with more complex dynamics, which require tight quantitative control or tuning.

## 1 Introduction

At present, genetic circuit design often involves multiple iterations of circuit optimisation aimed at addressing non‐functional interactions, handling unpredicted non‐orthogonality with the cell chassis, or fine‐tuning the dynamical parameters of the circuit. This is a lengthy and laborious process that usually requires years of optimisation to get to the final solution. One goal of synthetic biology is to apply rational engineering strategies to build synthetic gene circuits. Ultimately, this involves the assembly of modular genetic parts, closely described by robust quantitative dynamical models, into systems with predictable dynamic behaviours. Synthetic biology is already focusing on the development of genetic components with predictable quantitative behaviour [1, 2]. For example, a recent study describes a palette of modular genetic components for the assembly of genetic logic gates [1]. Furthermore, studies have also attempted to develop robust quantitative models to mimic the behaviour of simple genetic components and circuits [2]. The central aim of this study is to further research in this direction by developing and characterising a simple positive feedback genetic circuit module in *E. coli*.

Modelling has successfully guided the engineering of synthetic genetic systems in the past. For example, the seminal repressilator paper skillfully uses a deterministic model to evaluate the dependence of system behaviour on some of the key parameters, and to develop design rules for the desired behaviour [3]. A similar approach was adopted in the pioneering implementation of the toggle switch [4] and in that of the incoherent feed‐forward motif [5]. Furthermore, accurate models have been developed to predict the steady‐state activity of logic gate circuits [1], in one of the most intricate engineering attempts of synthetic biology to date. However, modelling has not yet been used to guide the quantitative tuning of a complex dynamical genetic circuit, which may be necessary to successfully engineer systems that rely on extensive and precise fine‐tuning. Turing patterns represent examples of such systems, where engineering precise periodic patterns have remained elusive to synthetic biologists to this day [6, 7]. This is likely due to the lack of quantitative models that tightly predict the behaviours of the underlying biological systems. The availability of well‐characterised synthetic components and the detailed study of the effects of their fine‐tuning on model parameters are likely to benefit the engineering of such systems. This study is a step in this direction and provides a complete, quantitative description of an intracellular positive feedback motif that can be implemented in larger circuits with more complex dynamic behaviour.

In this work, the synthetic gene circuit is built using filamentous M13 phage components. Holding a small genome of ∼10 kb that can easily be modified in vitro, releasing progeny without lysing the host cells, M13 phage offers sets of functionally orthogonal parts for the *E. coli* chassis and occupies a vital role in synthetic biology [8, 9, 10, 11]. The implementation of our positive feedback circuit is based on M13 phage gene IV protein (pIV) encoded by gene gIV, a membrane protein that is involved in phage assembly and secretion, which stimulates the phage shock response in response to its expression in *E. coli* [12]. The presence of pIV in the inner bacterial membrane leads to the recruitment of PspA, dissociation of the PspA‐PspF complex and PspF‐mediated induction of the *pspA* operon and *pspG* [13, 14, 15]. The positive feedback circuit built in this study consists of pIV expressed under the pspA promoter (Ppsp). The first part of the study develops, assembles and characterises a dual‐input Ppsp promoter variant, engineered to be repressible by TetR, similar to the one described in [16]. The positive feedback circuit is then assembled and characterised in liquid culture. Next, a simple ordinary differential equation model is fitted to the behaviour of the system at different levels of TetR repression. The fitting process is performed with a stepwise approach that allows the dissection of model parameters and dependencies between them. Aspects of this fitting approach are generalisable and may be applied to related parameter fitting problems in the future.

## 2 Results

### 2.1 Challenges of model fitting to time‐series in vivo data

**Fig. 1:**
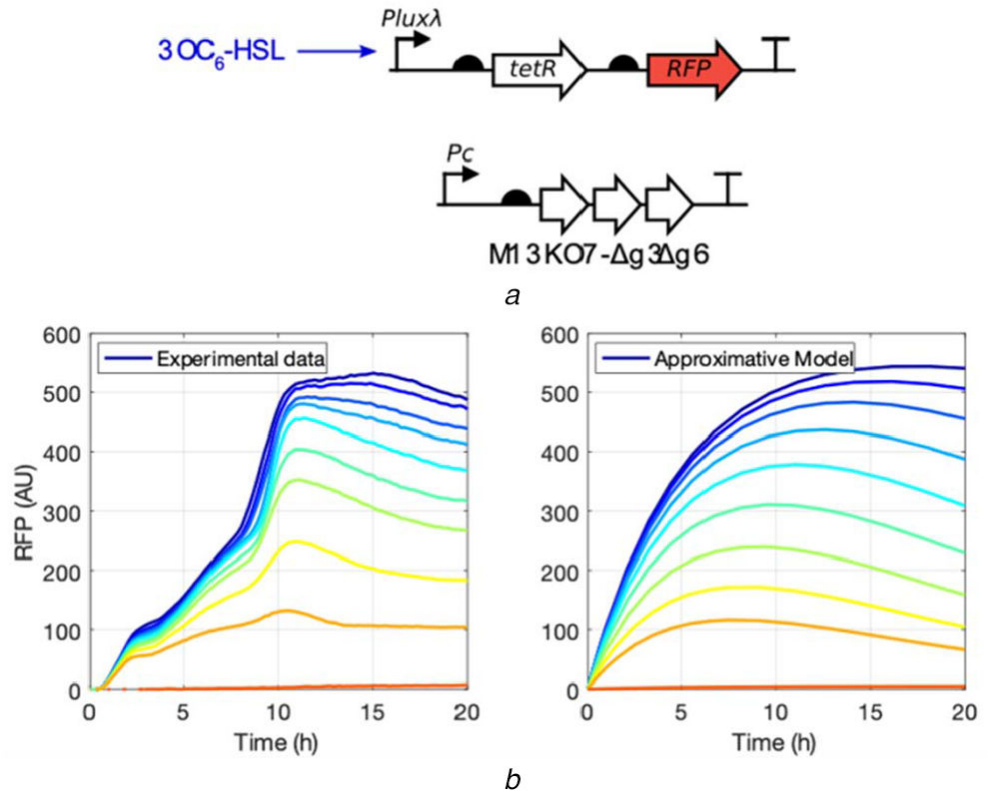
*Simple Pluxλ promoter system* [17] *induced with exogenously administered 3OC_6_‐HSL* ***(a)*** Diagram of the tested circuit. The circuit activity is measured in the context of phage gene expression from an M13KO7 phagemid helper plasmid variant lacking phage gIII, gIV and gVI [9, 18]. The phage components and TetR are present as part of a larger circuit engineering project and may contribute to the experimental behaviour deviating from an idealised model, ***(b)*** Experimental RFP expression data on the left and kinetic model that approximates the behaviour of the biological system on the right. Concentrations of 3OC_6_‐HSL are 0, 10^−2^, 2 × 10^−2^, 5 × 10^−2^, 10^−1^, 2 × 10^−1^, 5 × 10^−1^, 10^−0^, 2 × 10^−0^, 5 × 10^−0^ μM (from red to blue). The system was induced in early exponential phase and reaches the stationary phase at the 10 h timepoint (data not shown)

Fitting simple kinetic models to biological in vivo data is often a challenge due to the incongruence between the qualitative behaviour of the model and the underlying system. An example of such an incongruence is shown in Fig. 1, where a simple quorum‐sensing system is induced with increasing concentrations of exogenously applied 3OC_6_‐HSL and a fluorescent output is measured over time. The simple kinetic model used to describe the system shows convex, parabolic, saturating behaviour (for model definition, see Suppl. Text 1). This behaviour is not mimicked by the biological system which displays concave fluorescence accumulation curves. While the final fluorescence levels depend on the initial 3OC6‐HSL concentration, the rate of fluorescence increase seems to be only weakly dependent on the inducer's concentration for the stronger induction conditions (green and blue lines in Fig. 1*b*). Such behaviour could not possibly be captured by the simple model shown in Fig. 1*b*, regardless of the parameter combination.

Often, fitting the model requires adding complexity, by introducing more equations and parameters, to make the simulated behaviour qualitatively more similar to that of the underlying data [2]. While it often effectively leads to better fits, it can result in large and overparameterised models [6, 19], often based on poorly‐grounded assumptions. Thus, it is of interest to develop circuits described by simple, minimal models [19, 20]. Simpler models have higher predictive value and can more easily be reused as part of larger systems. This study develops one such circuit, well described by a single equation ODE model.

### 2.2 Part characterisation and system definition

To build a positive feedback loop, first, a hybrid dual‐input pspA promoter (Ppsp) was developed, that can be activated by pIV and repressed by TetR. In nature, the Ppsp promoter regulates the genetic response in *E. coli* to M13 phage infection. Upon infection, phage secretin pIV expression stimulates the phage shock response by causing the release of transcription factor pspF from the membrane, which binds to Ppsp and activates it [12, 13]. Five different Ppsp‐tetO (Ppspt) constructs were developed by inserting or substituting the tetO operator sequence into five different sites of Ppsp (Suppl. Fig. 1). The sequence of Ppsp is based on [16], whereas the design of the Ppspt variants is informed by Carlson *et al.* [8]. The Ppspt3 variant showed the highest dynamic range in terms of induction and repression and was thus selected for further analysis (Suppl. Fig. 2).

**Fig. 2:**
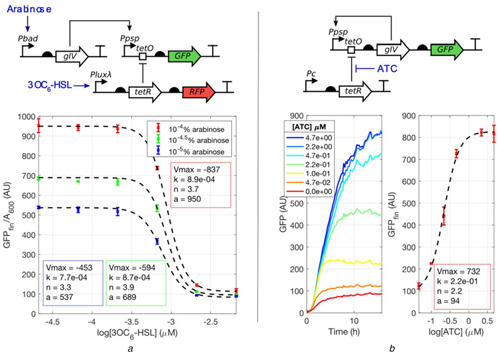
Ppspt3 characterisation and positive feedback system behaviour ***(a)*** Ppspt3 promoter is characterised in a circuit where gIV is placed under an arabinose‐inducible Pbad promoter and tetR is placed under a 3OC_6_‐HSL‐inducible Plux*λ* promoter. GFP is used as the Ppspt3 promoter‐reporter. Note that pIV is the protein product of gIV. The circuit was tested in the presence of 5.6 × 10^−2^ μM doxycycline to reduce the tone of background TetR inhibition due to leakiness of Plux*λ*. The plot shows fluorescence at 16 h after induction. The results show that Ppspt3 is inducible by arabinose‐mediated gIV expression. The results also show that Ppspt3 is repressible by 3OC_6_‐HSL‐mediated tetR induction. Coloured boxes show the parameters of inhibitory Hill function that best fit the data, ***(b)*** Positive feedback circuit consists of gIV placed under the Ppspt3 promoter. Its activity can be titrated with ATC, which derepresses constitutively expressed TetR. GFP is the positive feedback reporter. The plots show the time series and final timepoint measurements of GFP as a function of ATC concentration. All values are plotted as mean ± SEM (*n* = 3)

A detailed dose‐response study of Ppspt3 is shown in Fig. 2*a*. Two well‐characterised promoters were used to express the activating and repressive stimuli: inducer pIV was expressed from an arabinose inducible Pbad promoter [21], whereas the repressor TetR was expressed from the 3OC_6_‐HSL inducible Plux*λ* quorum sensing promoter [17]. The latter was also shown to be functional in Fig. 1. As expected, the data shows that Ppspt3 is activated by arabinose‐mediated pIV induction in the absence of 3OC_6_‐HSL stimulation. Additionally, Ppspt3 is repressed by TetR induction with 3OC_6_‐HSL. Inhibitory Hill functions were fitted to the GFP measurements (see Methods, (5)). Comparable half‐activation constants at the three arabinose concentrations show that repression kinetics are likely to be independent of the induction level of the promoter, hinting to a non‐competitive interaction between the activating and repressing stimuli. This interaction is further explored in Suppl. Fig. 3. Overall, the Ppspt3 promoter variant behaves as expected with respect to the activating and inhibiting stimuli and is suitable to be implemented in the positive feedback circuit.

The positive feedback circuit consists of pIV expressed under Ppspt3 (Fig. 2*b*). Anhydrotetracycline (ATC) can be used to disinhibit constitutively expressed TetR and regulate the activity of the circuit. Fluorescence measurements show that the positive feedback gradually turns on as TetR is disinhibited with increasing amounts of ATC (Fig. 2*b*). The relationship between final GFP levels and ATC concentration is fitted with a Hill function.

The positive feedback circuit is described with a system of two ODEs, the first describing the production rate of pIV and the second describing the binding equilibrium between TetR and ATC. Quasi‐steady state was assumed for the TetR‐ATC binding equilibrium because it occurs on a much faster time scale compared to pIV production. This reduces the system to a single equation with two positive production terms and a negative linear degradation term, where [*A*] is the activator pIV (1). All intermediate signalling steps that transduce the pIV stimulus to Ppspt3 promoter activation were abstracted. The purpose of this abstraction was to provide the simplest possible model: one that could easily be reused in other contexts and incorporated into larger models. Such an approach also prevents model over‐parametrisation and reduces the possibility of overfitting [19]. For further information on model derivation and parametrisation refer to Suppl. Text. 2. Briefly, the two assumptions used to derive the model are that: (i) the promoter state transitions occur at a much faster time scale than protein synthesis and degradation, and (ii) that mRNA production and degradation occur at a faster time scale than that of proteins. Hence, quasi‐steady‐state assumptions were applied for these two processes

(1) $$\begin{array}{rl} \frac{d\left[A\right]}{dt} & =b_{A}+V_{1}\left(\frac{{\left(\left[A\right]/K_{1a}\right)}^{n}}{1+{\left(\left[A\right]/K_{1a}\right)}^{n}}\right)\\ & \left(\frac{1}{1+\left((T_{\mathrm{tot}}/K_{t})/\left(1+{\left(k\left[\mathrm{ATC}\right]\right)}^{2}\right)\right)}\right)-\mu_{A}\left[A\right] \end{array}$$

The next sections aim to determine the parameters of this model, so that it best fits the time‐series data in Fig. 2*b*. The molecular species [*A*] is fitted to the GFP time course measurements, which are assumed to fully capture the quantitative behaviour of [*A*]. In other words, the levels of [*A*] are assumed to be directly and linearly proportional to the levels of GFP. The concentration of [*A*] will be expressed in terms of arbitrary units of GFP fluorescence.

### 2.3 Parameter fitting: activation terms

The approximate Bayesian computation (ABC) approach [22] was used to fit the models to the data. ABC is computationally expensive; however, it provides a good overview of the sampled parameter space, allowing one to easily determine if the fitting converges to a single solution or if multiple optima are possible. The model of (1) is parameterised by a total of seven parameters. In the first fitting attempt, all model parameters were fit simultaneously. The resulting parameter posterior distributions were not centred around single best fit values, indicating that the fitting process did not converge to a single solution (Suppl. Figs. 4, 5). The posterior distributions were difficult to interpret; that is why a stepwise fitting approach was developed to dissect these results and identify the redundant parameters or parameter dependencies that may have caused the fitting process not to converge.

The stepwise fitting approach relied on the observation that the model in (1) takes the form of (2) when the positive feedback is turned off and the TetR term is equal to zero. On the other hand, the model takes the form of (3) when the positive feedback is fully turned on and the TetR inhibition term is equal to one.

(2) $$\frac{d\left[A\right]}{dt}=b_{A}-\mu_{A}\left[A\right]$$

(3) $$\frac{d\left[A\right]}{dt}=b_{A}+V_{1}\left(\frac{{\left(\left[A\right]/K_{1a}\right)}^{n}}{1+{\left(\left[A\right]/K_{1a}\right)}^{n}}\right)-\mu_{A}\left[A\right]$$

**Fig. 3:**
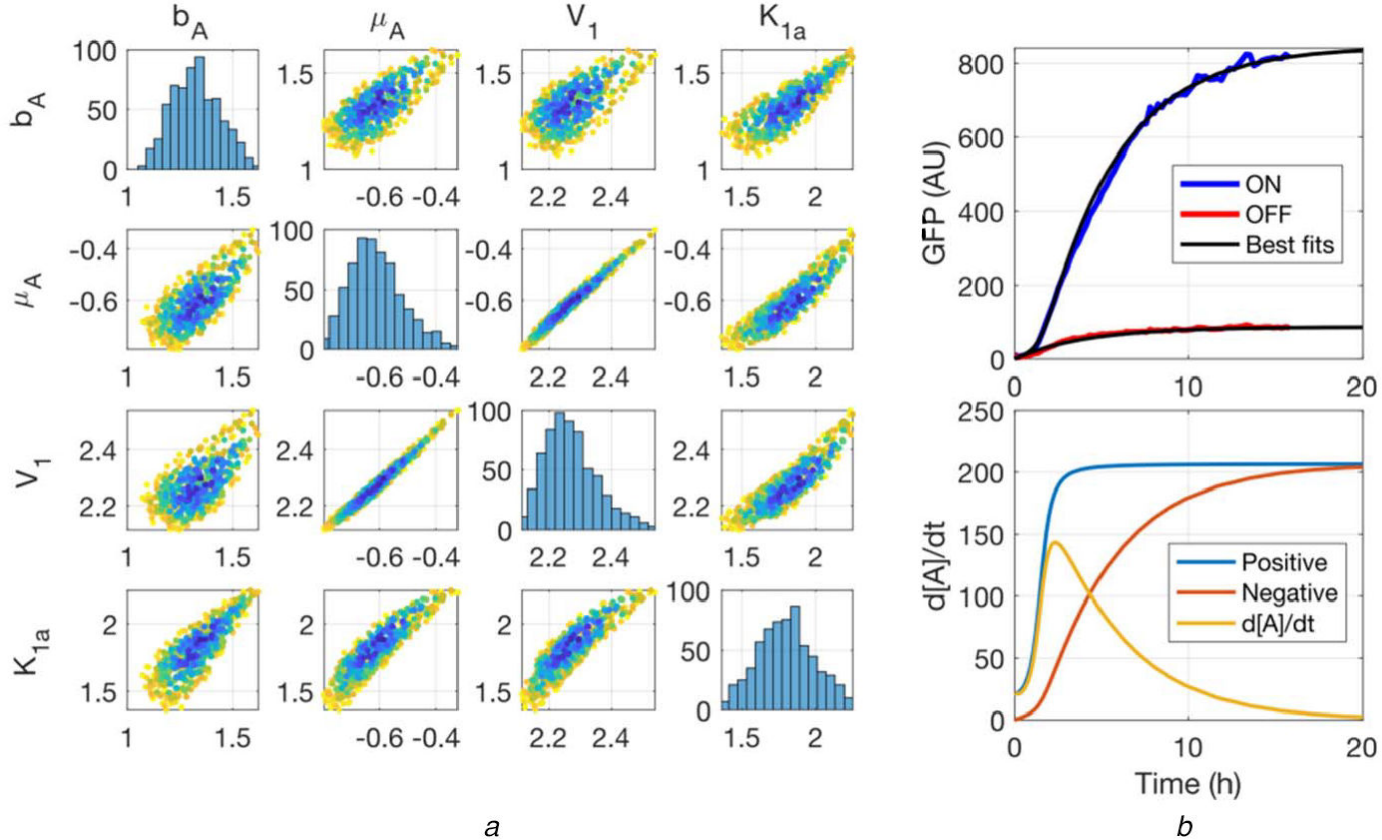
*Fit of* (2), *with n = 2, to the fully turned‐off condition and* (3) *to the fully turned‐on condition of the positive feedback circuit of Fig*. 2b ***(a)*** Posterior distributions for parameters *b_A_*, *μ_A_*, *V*_1_ and *K*_1*a*_, ***(b)*** Plots of the best fitting parameter combination, derived as the mean of the multivariate Gaussian fit to the posterior distributions shown in (a) (not shown). Upper panel shows the best fits of the time‐series data. The lower panel shows the rate profiles of the best fit, where ‘Positive’ refers to the positive terms of (3), whereas ‘Negative’ refers to the negative degradation term

The first step consisted of fitting parameters *b_A_* and *μ_A_* of (2) to the GFP measurements for the turned‐off condition in the absence of ATC. The fitting process converged to a single solution (Fig. 3). Note that for this simple model, the steady state of [*A*] equals the ratio between the background production and degradation rate parameters *b_A_*/*μ_A_*. The posterior distribution for *b_A_* and *μ_A_* is elongated along the straight dotted line that represents the *b_A_*/*μ_A_* ratio derived from the data, indicating that the fit is ‘sloppy’ along this line and that there may be a mild dependency in the best fits of the two parameters. However, the time scale of the early response allows the estimation of *b_A_* and *μ_A_* as a single best fit combination (Fig. 3*b*). The best fit values for the two parameters can be extracted as the mean of the multivariate Gaussian that best fits the posterior distributions.

**Fig. 4:**
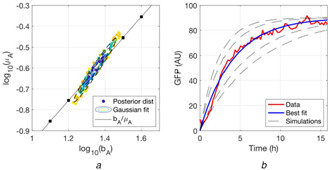
*Fit of* (2) *to the fully turned‐off positive feedback circuit shown in Fig*. 2*b* ***(a)*** Posterior distributions. The best‐fitting bivariate Gaussian distribution is shown in a dashed black contour plot. The mean of the Gaussian for *b_A_* is 22.1, whereas the mean for *μ_A_* it 0.246. The black line is the *b_A_*/*μ_A_* ratio as derived from the data; the black dots on the line mark the parameter combinations for which simulations are plotted with dashed black lines in (b), ***(b)*** Shows the data and the best fit, as derived from the mean of the bivariate Gaussian fit to the posterior distributions in (a)

Now that the simple background production and degradation model was successfully fitted to the turned‐off condition, the other parameters were introduced gradually to identify the point of failure of the fitting process. In the next step, the models of (2) and (3) were simultaneously fit to the fully‐off and fully‐on conditions, respectively. Initially, the cooperativity parameter *n* was fixed to one. The fitting process converged to single solutions with respect to the four parameters *b_A_*, *μ_A_*, *V*_1_ and *K*_1*a*_. Interestingly, equally good fits could be found when the fitting process was repeated for cooperativities *n* of two, three and four, indicating that the cooperativity parameter is sloppy (Supp. Figs. 6, 7, 8). The redundancy with respect to *n* is the first reason why the fitting process did not converge to a single solution when all parameters were fit simultaneously. The best fit for cooperativity of two and the associated posterior distributions are shown in Fig. 4. The posterior distributions of *V*_1_ and *μ_A_* are most elongated, indicating that the fit is somewhat ‘sloppy’ with respect to the ratio between these two parameters. Similarly, as in the first step of the fitting process, the behaviour of the model at early timepoints is expected to fix *V*_1_ and *μ_A_* at well‐defined values. The best fits for the four parameters were extracted by fitting a multivariate Gaussian. Derived parameters fitted the data well. The rate profile of the model shows an initial steep increase in the rate of production of [*A*] followed by an increase in its rate of degradation. As the steady state is approached, the rates of production and degradation tend to equality.

**Fig. 5:**
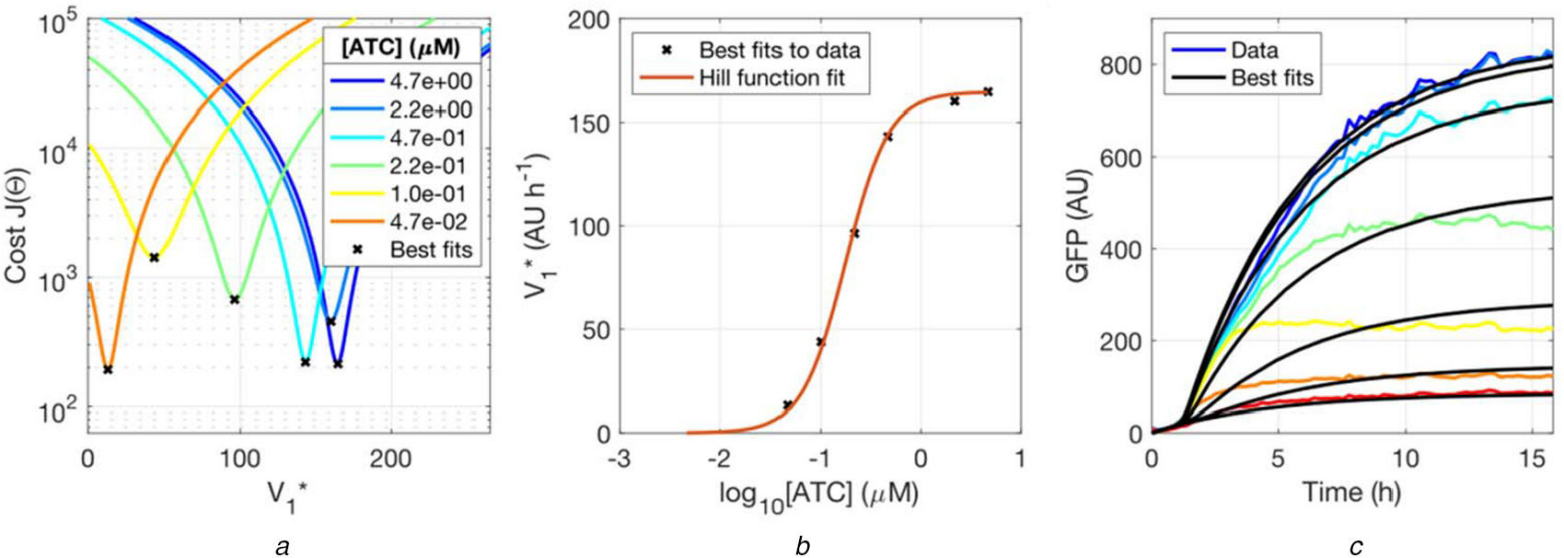
*Fit of the inhibitory term of* (4) *to the intermediate ATC concentrations* ***(a)*** Fitting of (4) parameter $V_{1}^{*}$ to the intermediate ATC concentrations. The figure shows the difference between model and data computed with the least squares cost function *J*(Θ) for a range of $V_{1}^{*}$ values. The best‐fitting $V_{1}^{*}$ that minimises each condition is highlighted with a black cross. The colours in (a) and (c) correspond, ***(b)*** The relationship of (4) where the best‐fitting $V_{1}^{*}$ value is plotted against ATC concentration. The *T*_tet_/*K*_t_ parameter of (4) is estimated to find the best fit, while *k* is fixed to 1.26 × 10^6^ μM^−1^. The black crosses between plots (a) and (b) correspond, ***(c)*** Plots of the data, as shown in Fig. 2*b* overlaid with the best fits

### 2.4 Parameter fitting: inhibition terms

The last step consisted of fitting the parameters associated to the TetR inhibition term of (1), *T*_tot_/*K_t_* and *k*. This term is a sigmoid function independent of [*A*]; it ranges between the values of zero and one, and scales *V*_1_ as a function of ATC concentration (4). This scaled *V*_1_ is denoted as $V_{1}^{*}$. $V_{1}^{*}$ ranges between zero and *V*_1_; it is zero when the system is completely turned off and it is *V*_1_ when the system is fully on

(4) $$V_{1}^{*}=V_{1}\left(\frac{1}{1+\left((T_{\mathrm{tot}}/K_{t})/\left(1+{\left(k\left[\mathrm{ATC}\right]\right)}^{2}\right)\right)}\right)$$

The reduced model of (3), where *V*_1_ is replaced by $V_{1}^{*}$, was fitted to each ATC condition independently; the fit was performed only with respect to $V_{1}^{*}$, the other parameters were fixed to the best fits identified in the previous steps of the fitting process (Fig. 5). Fig. 5*a* shows the values of $V_{1}^{*}$ that minimise the least‐squares cost function. The best fits for $V_{1}^{*}$ were plotted against ATC concentration essentially deriving the relationship described in (4) (Fig. 5*b*). The last step consisted of fitting parameters *T*_tot_/*K_t_* and *k* of (4) to the relationship between $V_{1}^{*}$ and ATC. The fitting showed that there is a linear dependency between parameters *T*_tot_/*K_t_* and *k* (Supp. Fig. 5). In addition to the cooperativity *n*, this was the second source of redundancy in the model parameters and additionally contributed to the lack of convergence of the initial fitting attempts, where all parameters were fitted simultaneously. It was reasoned that the binding affinity of ATC to TetR is relatively independent of the experimental context, so it was assigned the value of 1.26 × 10^6^ μM^−1^ drawn from the literature [23]. In this way, the value of *T*_tot_/*K_t_* could be identified and the fitting process brought to completion (Fig. 5*c*). The same process was also repeated to fit the models with cooperativities *n* of two, three and four, and equally good fits could be found in all the cases (Suppl. Fig. 9). The four models show equivalent dynamic behaviour, as shown by the simulations where ATC concentration was subjected to step function stimuli or oscillatory modulation (Supp. Fig. 10). Thus, they appear to be quantitatively equivalent.

### 2.5 Single‐cell measurements and stochastic model

**Fig. 6:**
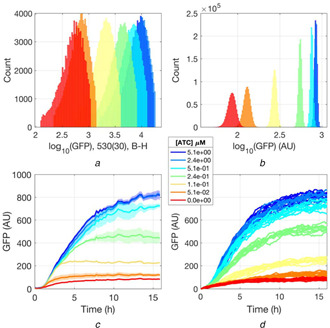
Single‐cell GFP fluorescence data and stochastic simulations of the positive feedback circuit ***(a)*** Distributions of single‐cell GFP fluorescence as a function of ATC concentration, quantified with flow cytometry, ***(b)*** Distributions of [*A*] concentration derived from the fluctuations of the stochastic model after it reaches a plateau (*t* > 100 h). Both (a) and (b) plot 10^5^ events, ***(c)*** Positive feedback circuit data in response to different concentrations of ATC, as shown in Fig. 2*b*, plotting the mean ± SEM (*n* = 3), ***(d)*** Ten simulations of the stochastic model of (5) are performed for each ATC condition. The best‐fitting parameter combinations for cooperativity *n* = 2 are used (Table 1)

The presence of positive feedback yield non‐linear circuits with bistability [24, 25, 26]. Circuit bistability can lead to multimodal distributions of single‐cell reporter expression levels. The positive feedback circuit of this study was assayed for bistability by measuring single‐cell GFP expression levels with flow cytometry. The flow cytometry assay was performed with fixed *E. coli* at the 16 h timepoint. Consistent with the population data, the single‐cell data shows an increase in fluorescence as a function of ATC concentration (Fig. 6*a*). The fluorescence distributions are monomodal and there is no evidence of bistability. The lack of bistability is consistent with the behaviour of the positive feedback model of (1), with a steady‐state analysis showing no bistability for the best fitting parameters (Table 1), for any value of cooperativity between one and four (Suppl. Figs. 11, 12). The analysis shows that a bistable circuit could be engineered by increasing *V*_1_ and *μ_A_* parameters (Suppl. Fig. 12). Increasing *V*_1_ could potentially be achieved by strengthening the ribosome binding site on gIV, whereas *μ_A_* could be increased by tagging it for degradation. These mathematical analyses illustrate the strength of the developed quantitative model in predicting circuit behaviour. These predictions should be tested in future enquiries for more definitive evidence of the utility of the model.

**Table 1:** Parameter values of the best fits to the data with respect to different cooperativities of the induction function

	*b_A_*, AU h^−1^	*V*_1_, AU h^−1^	*K*_1*a*_, AU	*μ_A_*, h^−1^	*T*_tot_/*K_t_*, h^−1^
*n* = 1	25.6	305.0	184.9	0.327	3.20 × 10^10^
*n* = 2	20.7	185.1	58.25	0.242	5.52 × 10^10^
*n* = 3	19.8	172.2	46.43	0.226	5.79 × 10^10^
*n* = 4	18.1	164.5	32.8	0.214	5.80 × 10^10^

The final step involved simulating the parameterised positive feedback circuit model of (1) in the presence of noise and attempting to mimic the flow cytometry data. A stochastic differential equation was defined by introducing a Gaussian white noise *ξ*(*t*) term. The amplitude of the noise is given by the square root of the sum of all the positive terms of (1) and by the sum of all the negative terms and is thus proportional to the rate of protein production and decay [27]. The Gaussian white noise has zero mean 〈*ξ*(*t*)〉 = 0 and auto‐correlation function of 〈*ξ*(*t*)*ξ*(*t′*)〉 = *δ*(*t* − *t′*), where *δ* is the Dirac delta function and angular brackets are population averages.

Figs. 6*c* and *d* show that the results of the stochastic simulations resemble the time‐course data. Secondly, the distributions of the fluorescence fluctuations in the plateau region (*t* > 100 h) mimic the monomodal increase in GFP expression levels seen in the flow cytometry data (Figs. 6*a* and *b*). The simulated distributions are, however, narrower than the measured distributions. One explanation for this may be that the many sources of noise related to the microscopic processes of transcription, translation and genetic regulation [28] are neglected in the present analysis; only noise on [*A*] is considered.

## 3 Discussion

The development of quantitative dynamic models that reliably describe the behaviour of synthetic genetic circuits and components is of crucial importance in enabling the application of rational engineering approaches to Synthetic Biology [2, 29]. In this study, a positive feedback circuit was first developed from simple genetic components. The circuit was characterised in *E. coli* liquid culture, and a simple single‐equation ODE model was built to describe its behaviour. Importantly, the seven model parameters could not be reliably identified by fitting them simultaneously to the circuit's time course data. Therefore, a step‐by‐step fitting approach was developed to overcome this problem, and this successfully fitted all model parameters and identified the sources of the model's redundancy. It was shown that the cooperativity of the activator is redundant: equally good fits can be found for multiple cooperativity values. It was also shown that a strong linear dependence exists between the binding affinity coefficient of ATC to TetR and that of TetR to its operator. The study characterises a positive feedback genetic circuit that can be reused in other synthetic systems, fully parameterises a minimal model that replicates its quantitative behaviour *in silico*, and develops a model‐fitting approach that can be applied to related model fitting problems. The last section of this study highlights the power of the quantitative model in guiding the tuning of the circuit to achieve a desired dynamic behaviour.

The model was observed to be redundant with respect to (i) the cooperativity of the activation function *n*, and (ii) the relationship between the inhibitory binding affinity parameters *T*_tot_/*K_t_* and *k*. This redundancy means that the model shows the same dynamic behaviour for more than one parameter combination. The redundancy of the inhibition parameters is resolved by drawing ATC's binding affinity to TetR from the literature [23]. Analogously, it may be possible to fix the activation cooperativity by quantifying the activator's degradation in a decoupled system and inferring from the relationship between cooperativity and degradation rate. It is likely that this cooperativity is greater than one because of the potentially cooperative hexamerisation process of the transcription factor pspF, involved in transducing the pIV signal to Ppsp [30, 31]. Precise parameter inference may, however, not be necessary. Computational studies show that some degree of redundancy is hard to avoid in systems biology models and argue that the predictive power of a model is more important than its exact parametrisation [32]. It is here shown that the described model's behaviour is qualitatively and quantitatively consistent among the redundant parameter combinations; thus, any parameter combination may be chosen to run circuit simulations.

Upon careful observation it may appear that the fit is slightly worse for early timepoints at intermediate ATC concentrations. The initial rate of fluorescence increase appears to be somewhat independent of ATC concentration, a behaviour that is not consistent with a simple mass action model, such as the one considered in this study. Such a behaviour could have resulted from the many interactions in the phage shock response pathway linking pIV expression to Ppsp activation [15, 31], which were omitted from the model. Upon careful consideration, it was decided not to expand the model further with the aim to preserve its simplicity and versatility to be used as part of larger models.

This study also develops a tuneable phage component that could potentially be applied to systems requiring tight tuning of the phage production rate, such as continuous evolution systems or reaction‐diffusion systems [6, 8, 9]. This could be achieved by combining our positive feedback system with an M13KO7 phage helper with a gIV deletion, and quantifying the relationship between pIV expression levels and rate of phage production. The circuit could also be applied to systems that rely on intracellular positive feedback loops, such as bistable toggle switches or oscillators [33, 34]. Filamentous M13 phage does not lyse the cell during its life cycle [35], potentially making it a suitable diffusible component to build reaction‐diffusion circuits [36].

## 4 Conclusion

The future of Synthetic Biology relies on the development of genetic components with predictable and fine‐tuned dynamic behaviours. The study develops and characterises a simple intracellular positive feedback circuit and designs a simple deterministic ordinary differential equation model that closely predicts its function. The developed components have the potential to be used in future engineering attempts that require tight quantitative control and fine‐tuning.

## 5 Methods

### 5.1 Strains and media

TG1 *E. coli* (Lucigen) with genotype [F’ traD36 proAB lacIqZ ΔM15] supE thi‐1 Δ(lac‐proAB) Δ(mcrB‐hsdSM)5(rK‐mK) were used for liquid culture experiments. TOP10 *E. coli* was used for molecular cloning. LB broth (Sigma‐Aldrich L3022) and 2xYT broth (ThermoFisher Scientific 22712020, unless otherwise specified) were used to culture the cells. SOC medium was used to recover the cells after transformation. Bacteriological agar (Sigma‐Aldrich A5306) was added to the broth whenever required. TfbI consisted of a water solution of 30 mM potassium acetate, 100 mM rubidium chloride, 10 mM calcium chloride dihydrate, 50 mM manganese chloride and 15% (v/v) glycerol; pH was adjusted to 5.8 with 10% (v/v) acetic acid. TfbII consisted of a water solution of 10 mM MOPS, 75 mM calcium chloride, 10 mM rubidium chloride and 15% (v/v) glycerol, pH was adjusted to 6.5 with dilute sodium hydroxide.

### 5.2 Liquid culture fluorescence assays

Electrocompetent TG1 *E. coli* were transformed by electroporation and plated onto LB agar plates with the appropriate antibiotics. Single colonies were cultured in 2xYT media (Sigma‐Aldrich Y1003) with appropriate antibiotics, incubated at 37°C in a Stuart SI500 orbital shaker (220 rpm) and grown to stationary phase for ∼16 h. The culture was transferred to a 96‐well plate (Greiner 655090) and 10 µl of each inducer dissolved in 2xYT media (Sigma‐Aldrich Y1003) was added to a final volume of 150 µl. Measurements of absorbance at 600 nm, GFP and RFP fluorescence were taken with the Tecan f200pro multimode microplate reader with the following settings: 37°C, 16 h kinetic cycle with 10 min kinetic interval, orbital shaking (2 mm amplitude, 280 rpm frequency), absorbance at 595/10 nm, GFP excitation at 485/20 nm, GFP emission at 535/25 nm, GFP gain of 20, GFP mirror Dichroic 510 nm, RFP excitation at 590/20 nm, RFP emission at 625/35 nm, RFP gain of 38, RFP 50% mirror, fluorescence top reading, fluorescence integration time 20 μs.

### 5.3 Flow cytometry

Immediately after the time‐course experiment, cells were fixed in 1% paraformaldehyde. Fluorescence and forward/side scatter were recorded on a BD LSR Fortessa cytometer. Cells were excited with the blue laser (488 nm), fluorescence was recorded with an emission filter of 530 (30) nm. The height of the signal peaks was considered, and 10^5^ events were measured for each ATC concentration. The data was analysed with MATLAB R2019a (Mathworks). The data was imported with the *fca_readfcs* function v. 2020.04.11 published on MATLAB File Exchange by Laszlo Balkay in April 2020. The outliers below the 5th percentile and above the 95th percentile were excluded from the analysis.

### 5.4 Electroporation of *E. coli*

Plasmid DNA (0.5 μl at 10 ng/μl) was transferred to an Eppendorf tube and cooled on ice. Cells were electroporated in a 0.1 cm gap cuvette (Bio‐Rad 1652089) with a 1.8 kV, 200 Ω, 25 mF pulse (Bio‐Rad Gene Pulser II Electroporation System). It was ensured that cells were kept cold throughout the electroporation procedure. The electroporated cells were transferred to 500 μl prewarmed SOC medium, incubated shaking at 37°C for 1 h and plated on LB agar with the appropriate antibiotics.

### 5.5 Making electrocompetent *E. coli*

TG1 *E. coli* were streaked onto LB agar with no antibiotic and incubated at 37°C overnight. A single colony was grown up in 2 mL of 2xYT medium overnight, transferred to 250 ml pre‐warmed 2xYT the following morning and incubated at 37°C to early exponential phase (OD600 of 0.4–0.6). All centrifugations steps were performed at 4000*g* for 5 min at 4°C unless otherwise specified. The culture was cooled on ice for 5 min and centrifuged. Cells were resuspended in 200 ml cold MilliQ water and centrifuged. Cells were resuspended in 100 ml cold MilliQ water and centrifuged. Cells were then resuspended in sterile 10% (v/v) glycerol (Sigma‐Aldrich G5516) and centrifuged for 7 min. The supernatant was discarded, and the pellet was resuspended in the solution remaining in the tubes. 100 μl aliquots were snap‐frozen on dry ice and stored at −80°C.

### 5.6 Molecular cloning

Q5 Hot‐Start High‐Fidelity DNA polymerase (NEB M0493) was used to amplify DNA fragments. All PCR reactions were set up using protocols provided by the manufacturers. Ligations of two or more DNA fragments were performed with the Gibson assembly HiFi NEBuilder kit (NEB E2621). DNA fragment preparation for Gibson assembly consisted of digesting with DpnI (NEB R0176), performing gel extraction from a 1% (w/v) agarose gel, run at 90 V for 1 h, with the Monarch DNA gel extraction kit (NEB T1020), assembling the purified fragments with the HiFi NEBuilder kit and transforming TOP10 *E. coli* with the chemical transformation protocol. The KLD enzyme mix (NEB M0554) was used whenever single DNA fragment ligation was required. After TOP10 transformation, positive clones were identified by colony PCR performed with Taq DNA polymerase with standard Taq buffer (NEB M0273). DNA was purified with the QIAprep Spin Miniprep kit (Qiagen 27106). All newly cloned constructs were sequence verified (Eurofins Genomics).

Detailed cloning protocols and primer design is omitted. Complete sequences of all genetic parts and plasmids are provided in the Supplementary Information. M13 phage gIV was cloned from the M13KO7 helper phage (NEB N0315S).

### 5.7 TOP10 competent cells and transformation

TOP10 cells were grown in 100 ml 2xYT media and allowed to reach an OD_600_ of 0.4. All centrifugations steps were performed at 3000*g* for 5 min at 4°C. The cells were cooled on ice for 15 min and centrifuged. The pellet was resuspended in 40 ml TfbI incubated on ice for 15 min then centrifuged. The cells were resuspended in 4 ml TfbII incubated on ice for 15 min and aliquoted on dry ice and stored at −80°C.

For transformation, 25 μl TOP10 competent cells were added to 1 μl DNA from previously performed assembly reactions, incubated on ice for 30 min, heat‐shocked at 42°C for 30 s and cooled on ice for another 2 min. Cells were recovered in 250 µl SOC media at 37°C with shaking incubation for 1 h. The cells were plated on LB agar with appropriate antibiotics and incubated at 37°C.

### 5.8 Data analysis and plotting

MATLAB R2019a (Mathworks) was used to analyse data. Hill functions (5) were fitted to the final fluorescence measurements, which were isolated from the time‐series data. The Hill equation is expressed in terms of inducer [*X*]. Parameters include the background expression levels (*a*), the maximal induction levels (*V*_max_), the Michalis‐Menten constant (*k*) and cooperativity (*n*). The DNA plotting Python library DNAplotlib was used to generate figures of the genetic circuits [37].

(5) $$f\left(\left[X\right]\right)=a+V_{\text{max}}\left(\frac{{\left[X\right]}^{n}}{k^{n}+{\left[X\right]}^{n}}\right)$$

### 5.9 Parameter fitting with ABC

MATLAB R2019a (Mathworks) was used to perform the model fitting. The ABC fitting process [22] consisted of these steps: (1) the parameter combination was sampled from the prior distributions; (2) the *ode15s* solver was used to simulate the model with the sampled parameters; (3) the least squares formula was used to calculate the square of the difference between the model simulation and the data; (4) the process was iterated multiple times; the number of iterations ranged between 1 × 10^7^ and 5 × 10^7^. All parameters besides the cooperativities were sampled from log‐uniform distributions; cooperativities were sampled from discrete uniform distributions of positive integers. Bounds of the prior distributions were chosen to be broad and were defined arbitrarily; no prior knowledge was used. For prior distribution definitions see Suppl. Text 6. The posterior distributions were derived by plotting the parameter combinations that give fits for which the least‐squares optimisation function is lower than an arbitrarily defined threshold. The values of the best fitting parameter combinations were extracted from the posterior distributions as the means of a Gaussian mixture model with no mixing estimated with the *fitgmdist* in‐built MATLAB function.

### 5.10 Stochastic model simulations

Simulations of the stochastic differential equation model were performed with an in‐house Euler solver implemented in MATLAB R2019a (Mathworks). The Gaussian white noise *ξ*(*t*) was sampled with the *randn* normal random number generator, scaled by the noise magnitude term, divided by the square root of the timestep size and added to the differential equation. The noise magnitude is given by the square root of the sum of the positive and negative rate terms of the corresponding deterministic differential equation of (1).

As already mentioned in Section 2, the single‐cell fluorescence distributions were derived from the stochastic model simulations by simulating the model in the plateau phase for a long period of time for *t* > 100 h. The single‐cell distributions correspond to the distributions of the model's fluctuations in the plateau phase, once the response is fully developed.
